# Exploring Propylene Carbonate as a Green Solvent for Sustainable Lithium‐Ion Battery Cathode Manufacturing

**DOI:** 10.1002/cssc.202500937

**Published:** 2025-08-20

**Authors:** Mazedur Rahman, Hosop Shin

**Affiliations:** ^1^ School of Mechanical Engineering Purdue University Indianapolis IN 46202 USA

**Keywords:** cathode fabrications, distributions of relaxation time, green solvents, lithium‐ion batteries, N‐Methyl‐2‐pyrrolidone, propylene carbonate, sustainable

## Abstract

This study pioneers the use of propylene carbonate (PC) as a green solvent alternative to N‐methyl‐2‐pyrrolidone (NMP) for Li‐ion battery cathode manufacturing, addressing a critical gap in sustainable electrode fabrication. Unlike prior research focused on half‐cell evaluations of alternative solvents, this work uniquely extends to both half‐cell and full‐cell configurations across multiple cathode chemistries, offering a comprehensive assessment of PC's viability. Electrodes prepared using PC exhibit comparable coating quality and morphological characteristics, including uniform particle distribution and structural integrity, to NMP‐processed counterparts. Electrochemical assessments indicate that PC‐based electrodes provide superior first‐cycle Coulombic efficiency and closely match the electrochemical performance of NMP electrodes at lower active material (AM) loadings, including stable capacity retention and minimal polarization even at higher C‐rates. However, at higher AM loadings, the PC‐based electrode exhibits increased interfacial and contact resistance, possibly due to incomplete PC solvent removal, leading to reduced capacity retention and increased polarization at higher C‐rates. These limitations suggest that while PC is a promising sustainable alternative, its practical application requires further optimization, including refining drying processes to enhance solvent removal and interfacility stability.

## Graphical abstract

This study pioneers the use of propylene carbonate (PC) as a green solvent alternative to N‐methyl‐2‐pyrrolidone (NMP) for Li‐ion battery cathode manufacturing, addressing a critical gap in sustainable electrode fabrication. The results demonstrate PC's compatibility with polyvinylidene fluoride (PVDF) binders and its potential to support ecofriendly electrode fabrication with minimal compromise in electrochemical performance.

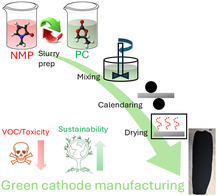

## 1 Introduction

The global transition to renewable energy systems has intensified the demand for sustainable lithium‐ion battery (LIB) manufacturing processes. Central to this challenge is the ubiquitous use of *N*‐methyl‐2‐pyrrolidone (NMP), a dipolar aprotic solvent, employed to dissolve polyvinylidene fluoride (PVDF) binders during electrode fabrication. While NMP enables homogenous distribution of active materials (AMs) across aluminum current collectors in LIB cathodes, its classification as a reproductive toxin by the European Union's Registration, Evaluation, Authorization, and Restriction of Chemicals (REACH) and designation as a hazardous air pollutant by the U.S. Environmental Protection Agency (EPA) has necessitated urgent alternatives.^[^ ^1^, ^2^ ^]^ This regulatory pressure aligns with the growing industrial awareness that NMP's energy‐intensive recovery process, which involves vacuum drying at high temperatures, contradicts net‐zero carbon commitments in battery manufacturing.

Water‐soluble binder systems, such as carboxymethyl cellulose and polyacrylic acid (PAA), initially emerged as promising alternatives to eliminate NMP from electrode fabrication, reducing costs and environmental impact.^[^ ^3^, ^4^ ^]^ However, their adoption introduces several challenges that hinder industrial scalability. The high surface tension of water impedes proper wetting of the electrode surface, resulting in uneven binder distribution on AM particles and poor adhesion to aluminum current collectors. Dispersants are required to mitigate particle agglomeration, yet these additives increase manufacturing complexity and cost.^[^ ^5^, ^6^ ^]^ Compatibility issues further limit their applicability. Hydroxyl group interactions in aqueous environments accelerate the degradation of Ni‐rich cathodes through lithium leaching, rock‐salt phase formation, and the formation of unwanted surface byproducts (e.g., LiOH and Li_2_CO_3_),^[^ ^7^, ^8^, ^9^, ^10^ ^]^ while corrosion of aluminum current collectors causes electrode cracking and compromises electrode structural integrity during cycling.^[^ ^8^, ^11^ ^]^ Overcoming these challenges necessitates the development of alternative solvents capable of dissolving PVDF without sacrificing electrochemical performance and material compatibility.

Recent investigations into green solvents for electrode manufacturing, such as triethyl phosphate (TEP) and dimethyl sulfoxide (DMSO), have revealed persistent trade‐offs between sustainability and functionality.^[^ ^12^, ^13^, ^14^ ^]^ While TEP effectively dissolves PVDF, it leads to inhomogeneous carbon distribution in the TEP‐processed electrode, increasing mechanical stress and negatively impacting cycling performance.^[^ ^12^, ^14^ ^]^ DMSO, despite its dissolution capability, introduces sulfur impurities and thermal instability above 40 °C, posing risks of hazardous emissions like SO_2_ and H_2_S, which diminish its environmental benefits.^[^ ^13^, ^15^, ^16^ ^]^

Other alternatives have also been explored. Dihydrolevoglucosenone (Cyrene) enables LiNi_0.8_Mn_0.1_Co_0.1_O_2_ (NMC811) cathode manufacturing but requires temperatures above 80 °C for effective PVDF dissolution.^[^ ^17^ ^]^ However, the resulting electrodes exhibit inhomogeneous surface morphology and cracking, leading to lower discharge capacity and necessitating further process optimization. γ‐valerolactone (GVL) has shown promise in slurry preparation, particularly in studies examining electrode thickness and drying temperature.^[^ ^18^ ^]^ Although PVDF dissolves within an hour at 60 °C, GVL‐processed electrodes suffer from poor adhesion and significant capacity fade at high discharge rates.^[^ ^18^ ^]^

Methyl‐5‐(dimethylamino)‐2‐methyl‐5‐oxopentanoate (PolarClean) and dimethyl isosorbide (DMI) dissolve PVDF at low concentration (1–3 wt%) but require prolonged drying (72 h) to prevent electrode cracking.^[^ ^19^ ^]^ Furthermore, residual solvent molecules trapped within the electrode structure contribute to inconsistent and suboptimal electrochemical performance, emphasizing the need for further investigations into solvent compatibility and electrode stability studies.^[^ ^19^ ^]^ Meanwhile, NMC811 pouch cells fabricated with 3‐methoxy‐N,N‐dimethylpropionamide (MDMPA) have demonstrated slightly improved electrochemical performance at lower temperatures and during long‐term cycling compared to those prepared with NMP. However, safety concerns remain, as the National Institutes of Health (NIH) has identified MDMPA as a potential respiratory and eye irritant.^[^ ^20^ ^]^

Recent studies have broadened the landscape of green electrode manufacturing by proposing novel processing strategies, comprehensively evaluating alternative green solvents, and introducing new environmentally benign options. Kim et al. advanced a water‐based slurry approach using kosmotropic salt solutions to stabilize PVDF and suppress interfacial reactions, achieving high‐performing NMC811 cathodes with excellent scalability and reduced environmental impact.^[^ ^21^ ^]^ Marshall et al. introduced 3‐Methyl‐2‐oxazoliidinone (JEFFSOL MEOX), which dissolves PVDF at accessible temperatures (40–50 °C), enabling slurry preparation and coating processes similar to those using NMP.^[^ ^22^ ^]^ Legar et al. systematically assessed several solvents, including N‐N’‐dimethylpropyleneurea (DMPU), as potential NMP replacements, identifying promising candidates for further investigation in green battery manufacturing.^[^ ^23^ ^]^

Despite recent advancements, the search for safer and more effective alternatives remains critical, as currently proposed green solvents still present trade‐offs in processability, electrochemical performance, and environmental impact. Notably, most prior studies have primarily focused on replacing NMP in NMC cathode slurry preparation and have been largely limited to half‐cell evaluations. However, half‐cell tests alone do not fully capture the complexities of real‐world battery operation, as they do not account for full‐cell interactions, long‐term cycling stability, and practical manufacturing constraints. To bridge this gap, further research is needed to validate the effectiveness of green solvents across a broader range of cathode chemistries, such as LiCoO_2_ (LCO), and LiFePO_4_ (LFP), while also conducting comprehensive electrochemical assessments in full‐cell configurations. Addressing these gaps will be essential for enabling the widespread adoption of green solvents in industrial battery electrode manufacturing.

This study explores propylene carbonate (PC) as a promising green solvent for LIB cathode manufacturing, aiming to overcome the limitations of existing alternatives. Although the solubility of PVDF in PC has been previously reported—particularly as a latent solvent under elevated temperatures—its practical application in electrode fabrication, particularly under industrially relevant conditions, remains underexplored. To the best of our knowledge, this study represents the first systematic investigation of PC as a viable replacement for NMP in cathode slurry processing, with comprehensive evaluations of slurry behavior, electrode morphology, mechanical integrity, and full‐cell electrochemical performance.

Compared to previously studied solvents, PC offers a unique combination of moderate solvating power for PVDF at process‐compatible temperatures, low toxicity, high thermal stability, and complete biodegradability. Additionally, its nonvolatile nature significantly reduces hazardous emissions, making it a safer and more sustainable option for large‐scale applications.^[^ ^24^, ^25^ ^]^ This work addresses critical gaps in the literature by demonstrating that PC enables effective slurry mixing and uniform electrode coating without requiring extreme processing conditions. Importantly, PC‐processed electrodes are rigorously evaluated using both half‐cell and full‐cell configurations, across multiple cathode chemistries including NMC and LCO, to confirm their adaptability and electrochemical viability.

## 2 Experimental Section

### 2.1 Materials

PC (>99.7%, anhydrous, Sigma Aldrich) and NMP (>99.5%, Sigma Aldrich) were used as received without further purification. Li_1.05_Ni_0.33_Mn_0.33_Co_0.33_O_2_ (NMC111, MSE Supplies) and LCO (>99.8%, Sigma Aldrich) were employed as cathode materials. PVDF (HSV 900) was used as the binder, while carbon black (CB) (C65, Imerys) was added as a conductive additive during electrode fabrication.

### 2.2 Binder Dissolution and Cathode Fabrication

**Figure 1:**
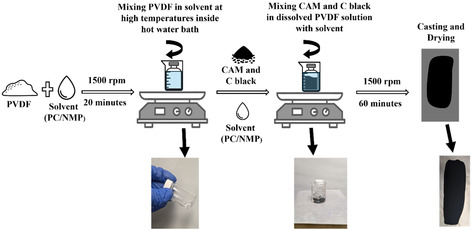
Schematic flowchart diagram for cathode fabrication.

**Figure** **1** depicts the schematic flowchart for cathode slurry preparation. Our previous works reported that PC acts as a latent solvent for PVDF, requiring temperatures of at least 80 °C for complete dissolution.^[^ ^24^, ^25^ ^]^ To ensure uniform heating and homogenous mixing, PVDF (8 wt%) was dissolved in PC using a hot water bath enclosure stabilized at 80 °C. The solution was stirred at 1500 rpm for at least 20 min or until a viscous, clear solution formed.

The (AMs) and CB were premixed by hand grinding, followed by further blending using a Thinky planetary mixer at 1000 rpm for 2 min. The dry mixture was then added to the binder solution in two sequential mixing steps of 30 min each. An additional amount of PC was added as needed to adjust the solution's viscosity. The final slurry was mixed for ≈1 h at 1500 rpm and then immediately coated onto Al foils using a doctor blade at room temperature.

For NMP‐based electrode fabrication, the same process was followed, except PVDF binder mixing was conducted at 55 °C. Two cathode compositions were prepared with AM, CB, and binder in weight ratios of 85:7.5:7.5 and 94:3:3. The coated NMC electrodes were initially dried at 100 °C on a hotplate, followed by overnight drying at 110 °C in a vacuum oven. The final aerial loading was ≈5 and 9 mg cm^−2^, respectively. Additionally, LCO electrodes were fabricated using the same process, with a composition of 94:3:3 and an aerial loading of 8 mg cm^−2^.

### 2.3 Slurry, Powder, and Electrode Characterization

The rheological behavior of cathode slurries was assessed using a rotary viscometer (CGOLDENWALL, NDJ‐5S). Slurry composition was fixed at 94:3:3 (wt%) NMC111:PVDF:Super P, with a total solid content of 35%. For the NMP‐based slurry, components were mixed at room temperature. For the PC‐based slurry, mixing was performed at 90 °C to facilitate PVDF dissolution. Rheology measurements were conducted immediately after mixing to minimize the effects of settling or phase separation. Viscosity was measured at four rotational speeds: 6, 12, 30, and 60 rpm, corresponding to approximate shear rates of 0.8, 1.6, 4.0, and 8.0 s^−1^, respectively. The data were fitted using the power‐law model.

(1) $$\eta =K{\dot{\gamma }}^{n-1}$$

where ***η*** is the viscosity, $\dot{\gamma }$ is the shear rate, *K* is the consistency index, and *n* is the flow behavior index. This model was used to characterize the shear‐thinning behavior of the slurries and assess their processability for mixing and coating applications.

To assess the mechanical integrity of the electrode coating and its adhesion to the aluminum current collector, a Kapton tape peel test was conducted using cathode samples prepared with dimensions of 50.8 × 20.3 mm^−2^. A strip of Kapton tape was applied to the electrode surface and pressed down firmly to ensure consistent contact. After a 10‐second dwell time, the tape was peeled off vertically at ≈90‐degree angle. The mass of the electrode was recorded before and after peeling to determine any material detachment.

A field‐emission scanning electron microscope (JEOL, JSM‐7800F) equipped with energy‐dispersive X‐ray spectroscopy (EDAX) was utilized to analyze the surface and cross‐sectional morphology of PC‐ and NMP‐processed electrodes. Thermogravimetric analysis (TGA) was conducted to assess the presence of residual solvent in dried electrodes. Measurements were performed using a Thermogravimetric analyzer (TA Instruments, SDT Q600) under nitrogen atmosphere to prevent oxidation. Powders scraped off from dried electrodes (≈7–10 mg) were placed in an alumina pan and heated from room temperature to 400 °C at a constant ramp rate of 10 °C min^−1^. Mass loss profiles were recorded as a function of temperature.

### 2.4 Cell Assembly and Electrochemical Measurements

For electrochemical testing, coin cells (CR2032) were assembled in an argon‐filled glovebox with oxygen and moisture levels maintained below 0.1 ppm. The cells were assembled in both half‐cell and full‐cell configurations, with lithium metal and premade graphite anodes (MTI Corp) as the counter electrodes, respectively. 1.2 M LiPF_6_ in a 1:1 mixture of ethylene carbonate and ethyl methyl carbonate was used as the electrolyte. After a 24‐hour resting period to ensure complete electrolyte wetting, cells underwent three formation cycles using a constant current (CC) charge/discharge protocol at a C/10 rate for half‐cells and C/20 rate for full‐cells, within a voltage range of 3.0–4.3 V. Following the formation cycle, electrochemical impedance spectroscopy (EIS) was conducted at a fully discharged state. EIS tests were conducted across a frequency range from 500 kHz to 0.1 Hz (half‐cell) and 0.01 Hz (full‐cell) with a 5 mV amplitude. Distribution of relaxation time (DRT) analysis was performed using DRT tools.^[^ ^26^ ^]^ Long‐term cycling performance was assessed over 100 cycles at a C/3 charge/discharge rate for both half‐cells and full‐cells. The rate capability of half‐cells was evaluated using a C/3 charging rate and different discharging rates, including C/3, C/5, 1C, 2C, and 3C. For the LCO electrode, only half‐cells were assembled and tested for demonstration purposes. These cells were formation cycled at a C/10 rate, followed by 100 cycles at a C/3 rate.

## 3 Results and Discussion

### 3.1 Sustainable Cathode Fabrication Using PC: Properties and Process

PC is a dipolar aprotic solvent characterized by low toxicity, high boiling and flash points, and low vapor pressure (**Table** **1**).^[^ ^27^ ^]^ These properties significantly enhance operational safety and reduce health hazards during cathode fabrication, aligning well with sustainable battery manufacturing goals.^[^ ^27^, ^28^ ^]^ Furthermore, PC's biodegradability contributes to a lower environmental footprint, offering a compelling advantage over traditionally used non‐biodegradable solvents.^[^ ^28^ ^]^

**Table 1:** Physical properties and HSPs for PVDF, NMP, and PC.^[^ ^31^ ^]^

Polymer/solvent	Boiling point [°C]	Flash point [°C]	Viscosity [mPa.s]	HSPs *δ*D, *δ*P, *δ*H [√MPa]
PVDF	–	–	–	17.2,12.5,9.2
NMP	204	91	1.7	18.0, 12.3, 7.2
PC	242	116	2.8	20.0, 18.0, 4.1

In cathode manufacturing, achieving solvent‐PVDF compatibility is essential for homogeneous slurry formation and uniform electrode microstructure upon drying.^[^ ^29^ ^]^ Despite its favorable environmental profile, PC demonstrates limited compatibility with PVDF at room temperature due to its latent solvent characteristics, requiring elevated temperatures for polymer chain disentanglement and dissolution.^[^ ^30^ ^]^ Hansen solubility parameters (HSP) confirm PVDF's limited solubility in PC under ambient conditions (Table 1).^[^ ^31^ ^]^ Our previous studies demonstrated effective dissolution of PVDF in PC at ≈80 °C, achieving comparable cathode recovery efficiencies to conventional solvents such as TEP and NMP.^[^ ^24^, ^25^ ^]^ Expanding on these findings, this study further explores sustainable cathode fabrication using PC as a PVDF binder solution.

Our study further confirms the critical role of temperature and uniform heating in achieving complete dissolution of PVDF in PC, which is essential for optimal slurry preparation. Experiments with PVDF concentrations (4 wt% and 8 wt%) at 80 °C using magnetic hotplate stirring alone for 24 h resulted in incomplete dissolution, evidenced by PVDF sedimentation within 10 min after the cessation of stirring and heating (Figure S1, Supporting Information). Conversely, uniform heating provided by a hot water bath enclosure facilitated rapid and complete polymer swelling and dissolution within 20 min, providing transparent PVDF solutions comparable to NMP‐based solutions (Figure S2, Supporting Information). This rapid dissolution significantly surpasses the prolonged dissolution times (>4 h) reported for alternative solvents like TEP, Cyrene, and DMSO.^[^ ^12^, ^13^, ^17^ ^]^ Upon cooling, PVDF solutions exhibited thermoreversible gelation typical of latent solvents, indicating reduced polymer mobility at lower temperatures (Figure S2, Supporting Information).^[^ ^18^, ^30^, ^32^ ^]^ Thus, preparing fresh PVDF solutions immediately prior to slurry mixing is crucial to avoid gelation‐induced viscosity variations and ensure optimal mixing.

**Figure 2:**
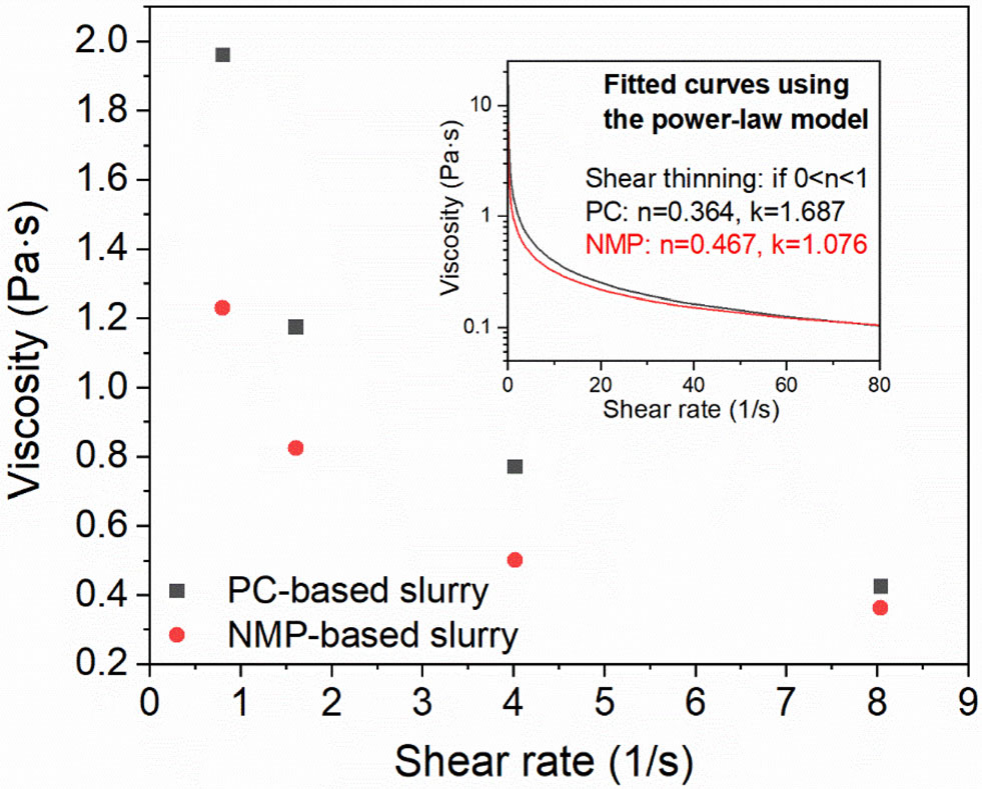
Viscosity versus shear rate curves for PC‐based and NMP‐based cathode slurries. The inset shows fitted curves using the power‐law model ($\eta =K{\dot{\gamma }}^{n-1}$), confirming shear‐thinning behavior for both systems.

An effective electrode slurry must exhibit shear‐thinning behavior to ensure uniform particle dispersion, minimize sedimentation, and enable consistent coating onto aluminum substrates.^[^ ^5^, ^33^ ^]^ To evaluate the processability of PC‐based slurries, viscosity was measured at multiple shear rates using a rotational viscometer and fitted using the power‐law model (**Figure** **2** and Table S1). Both PC‐ and NMP‐based slurries demonstrated shear‐thinning behavior with *n* < 1, characteristic of power‐law fluids, confirming their suitability for coating processes. At lower shear rates, PC‐based slurries exhibited slightly higher viscosity than NMP‐based slurries. However, as the shear rate increased, viscosity values converged, indicating that PC‐based slurries remain readily processable under shear conditions typical of industrial mixers.

**Figure 3:**
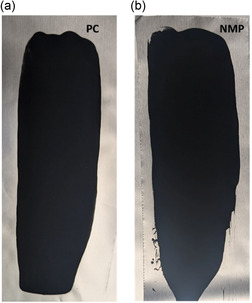
Photographs of dried NMC electrode coatings prepared using a) PC‐ and b) NMP‐based slurries. The electrode formulation was 85% AM, 7.5% CB, and 7.5% PVDF (by weight).

Visual observation further confirmed the excellent coating behavior of PC‐based slurries, similar to NMP‐based slurries. Optimizing the PC mixing conditions with an additional amount of the solvent resulted in desired slurries with high stability and no spreading during coating, comparable to NMP‐based slurries (Supplementary Video). Both PC‐ and NMP‐based electrodes displayed smooth coating and defined edges after drying, validating the applicability of PC as an NMP alternative (**Figure** **3**). Additional tests on LiCoO_2_ cathodes and higher AM content (94%) electrodes also demonstrated consistent coating quality with PC, underscoring its versatility across different chemistries and loadings (Figure S3, Supporting Information).

Electrode adhesion to the current collector is a key consideration in evaluating binder–solvent computability. A Kapton tape peel test was performed to assess the mechanical integrity of the electrode coating and its adhesion to the current collector (Figure S4, Supporting Information). The results indicated that PC‐processed electrodes maintained strong adhesion to the aluminum foil, with no significant delamination or material loss (less than 5%) after drying. This confirms that PC enables effective binder–collector interaction, resulting in mechanically robust electrode films.

**Figure 4:**
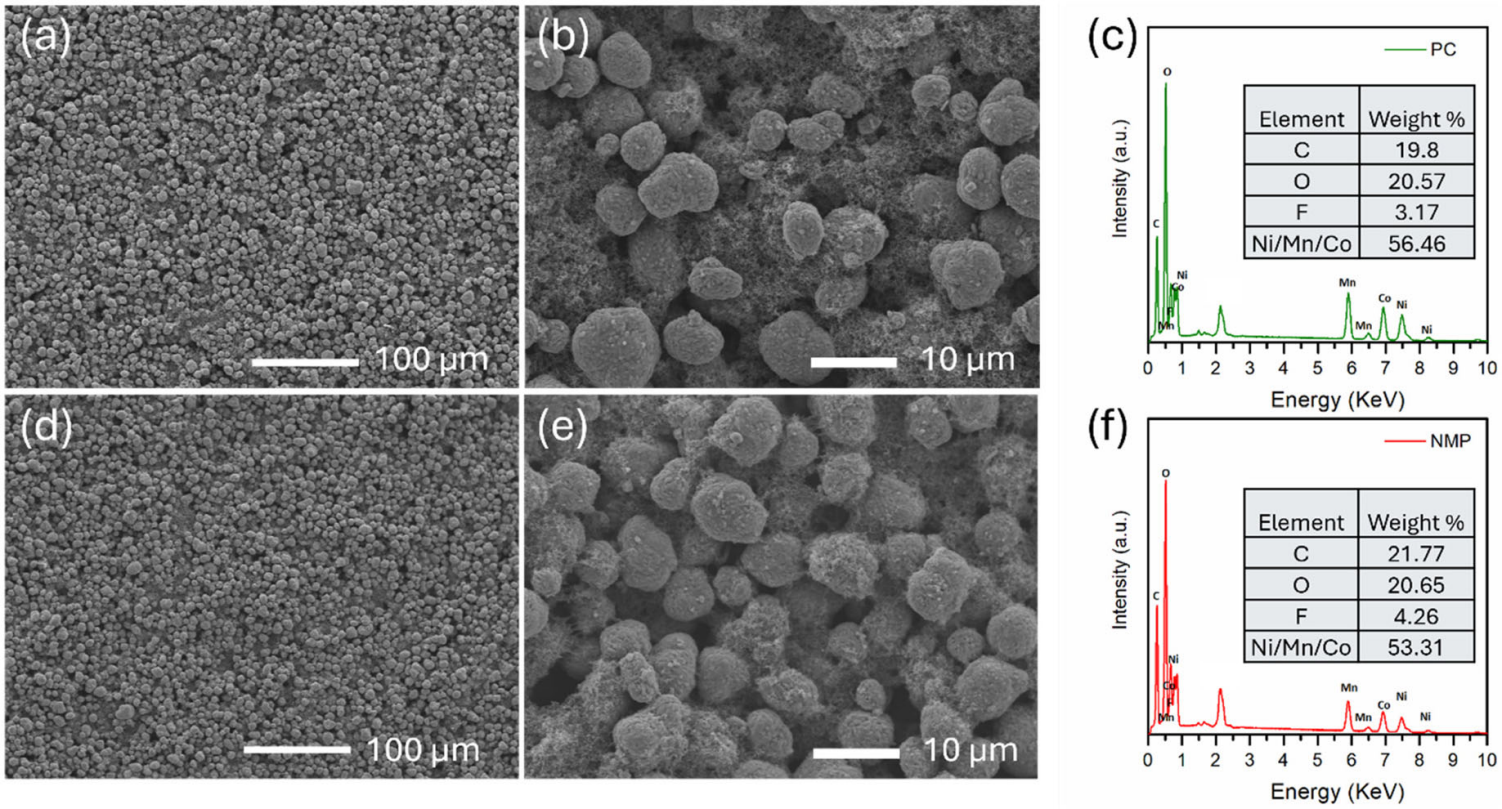
SEM images and corresponding EDAX spectra of a–c) PC‐ and d–f) NMP‐processed electrodes.

The SEM images (**Figure** **4** and Figure S5, Supporting Information**)** confirmed comparable secondary particle morphologies and uniformly distributed micrometer‐sized particles for both PC‐ and NMP‐based electrodes. In both cases, distinct and uniform porosity between secondary particles was clearly visible, a feature critical for electrolyte infiltration and wetting. No visible cracks or delamination indicated structural integrity. EDAX mapping further confirmed the uniform distribution of conductive carbon and binder networks across the cathode surfaces (Figure S6, Supporting Information).^[^ ^34^, ^35^ ^]^

To confirm binder distribution and assess the risk of migration during drying, we also performed cross‐sectional SEM imaging and EDX elemental mapping, using fluorine (F) as a marker for the PVDF binder (Figure S7, Supporting Information). The SEM images of both PC‐ and NMP‐processed electrodes showed dense and uniform particle packing across the electrode thickness with no signs of delamination or binder‐rich layers. The EDX maps further revealed a homogeneous distribution of fluorine, indicating that the binder remained uniformly distributed through the electrode during drying. These results suggest minimal binder migration under the tested fabrication conditions. While binder migration was not observed at the electrode thickness (≈35–45 μm), it is possible that thicker or denser electrodes could exhibit binder redistribution due to slower solvent removal. Further studies are warranted to evaluate binder behavior under such conditions.

### 3.2 Electrochemical Performance of PC‐Processed NMC Electrodes with Low Active Material Loading

Following the successful validation of morphological similarities between electrodes fabricated using PC‐ and NMP‐based binder solutions, we first evaluated their electrochemical performance in half‐cell configurations at low AM loading.

Notably, the PC‐processed electrode showed improved first‐cycle Coulombic efficiency (CE) (≈88%) compared to NMP‐processed electrodes and other green solvent‐based electrodes (Table S2).^[^ ^12^, ^17^, ^19^ ^]^ This enhanced first‐cycle CE is primarily attributed to lower first‐cycle charge capacity (Figure S8, Supporting Information), suggesting reduced irreversible capacity consumption during the first charge while maintaining a discharge capacity comparable to that of the NMP‐processed electrode. One possible explanation for the reduced first‐charge capacity is early surface passivation induced by residual PC, which may limit the number of available active sites for lithium deintercalation during the first charge while also helping to suppress parasitic side reactions.

Both NMP and PC are high‐boiling solvents that can be difficult to completely remove from electrodes under standard drying conditions. Previous studies have reported that conventional protocols often fail to fully evaporate NMP due to its low vapor pressure, unless aggressive drying conditions are employed.^[^ ^36^, ^37^ ^]^ As shown in the TGA analysis (Figure S9, Supporting Information), both NMP‐ and PC‐processed samples exhibited gradual mass loss up to 400 °C, with total weight loss remaining below 1%. However, the PC‐processed sample showed a slightly higher residual content, confirming that a portion of PC remains trapped within the electrode structure after drying. These residuals may undergo partial oxidative decomposition at high cathode voltages, contributing to early surface passivation and reducing further electrolyte decomposition.^[^ ^38^, ^39^, ^40^ ^]^

**Figure 5:**
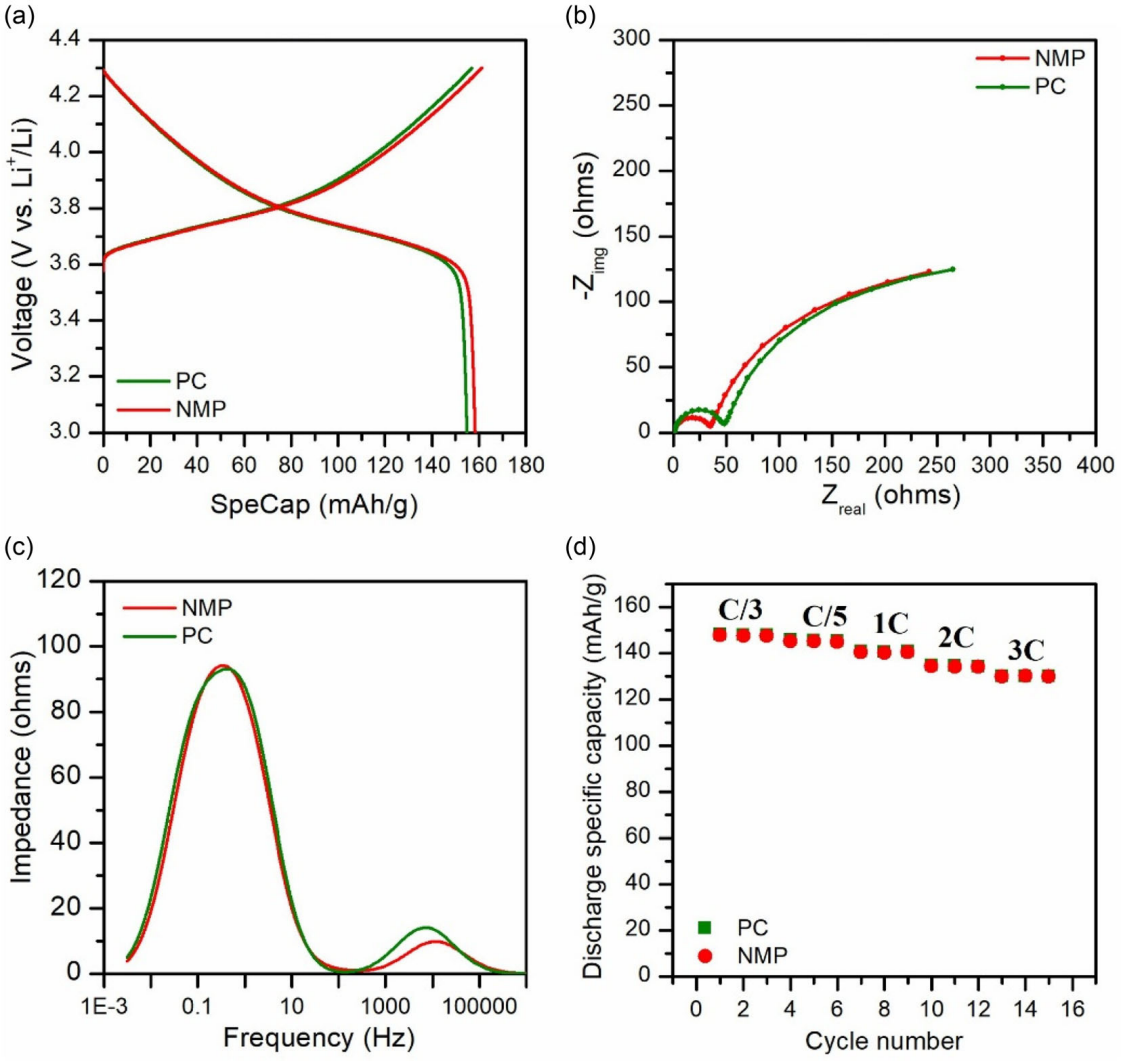
Electrochemical performance at low AM loading (85:7.5:7.5, AM/CB/PVDF, wt%) in half cells. a) Charge–discharge voltage profiles at the third formation cycle (C/10). b) Nyquist plots after formation. c) Corresponding DRT analyses highlighting impedance contributions. d) Rate capability tests showing capacity retention at varying C‐rates.

**Figure** **5**a presents the charge/discharge profiles of PC‐ and NMP‐processed electrodes at the third formation cycle. Both electrodes exhibited a typical voltage plateau around 3.7 V, consistent with the electrochemical behavior of NMC111, and delivered comparable specific discharge capacities at C/10. The minor difference in reversible capacity and the similarity in charge/discharge profile underscore the ability of the PC‐processed electrode to maintain electrochemical performance comparable to that of the NMP‐processed counterpart, while offering the additional advantage of environmental sustainability.

The Nyquist plots (Figure 5b) after the formation cycles indicated that the overall resistance remained quite comparable, although the PC‐processed electrode exhibited slightly higher interfacial resistance than its NMP counterpart. To further deconvolute the impedance contributions, DRT analysis was performed, as shown in Figure 5c. The main distinction between the two electrodes appeared in the high‐frequency DRT peak (>100 Hz), where the PC‐processed electrode showed a slightly more pronounced peak. This peak is typically associated with cathode–electrolyte interface (CEI) resistance and/or the contact resistance between the NMC particles and the current collector.^[^ ^41^, ^42^, ^43^, ^44^ ^]^ In contrast, the low‐frequency DRT peak (<10 Hz) was comparable for both electrodes, indicating similar charge transfer kinetics and solid‐state Li^+^ diffusion in the NMC particles.^[^ ^41^, ^42^, ^43^, ^44^ ^]^ This similarity in charge transfer kinetics and diffusion dynamics further supports the comparable electrochemical performance of the two electrodes at low AM loading.

Consistent with the DRT results, the PC‐ and NMP‐processed electrodes exhibited comparable rate capability, retaining over 87% of their initial capacities at 3C (Figure 5d), with CEs exceeding 99%. This similarity indicated that the slightly higher impedance observed in EIS and DRT analysis for the PC‐processed electrode has a negligible impact on rate performance under the tested conditions. The comparable electrochemical performance of PC‐ and NMP‐processed electrodes, combined with the improved first‐cycle CE of the PC‐processed electrode, highlights its potential as a sustainable alternative to NMP for cathode fabrication. Although PC requires slightly higher temperatures than GVL for PVDF dissolution, previously reported GVL‐processed electrodes suffered from substantial capacity degradation at high discharge rates.^[^ ^18^ ^]^

To further validate the versatility and viability of PC as an alternative to NMP across different cathode chemistries, we also fabricated PC‐processed LCO electrodes and evaluated their electrochemical performance in half‐cell configurations (Figure S10, Supporting Information). After the initial formation cycles, the PC‐based LCO electrode exhibited a discharge capacity of 153.7 mAh g^−1^, closely matching the manufacturer's reported value of 154 mAh g^−1^. Moreover, stable long‐term cycling performance was demonstrated, further confirming the broader applicability and reliability of PC‐processed electrodes across diverse cathode chemistries.

### 3.3 Electrochemical Performance of PC‐Processed NMC Electrodes with High Active Material Loading

**Figure 6:**
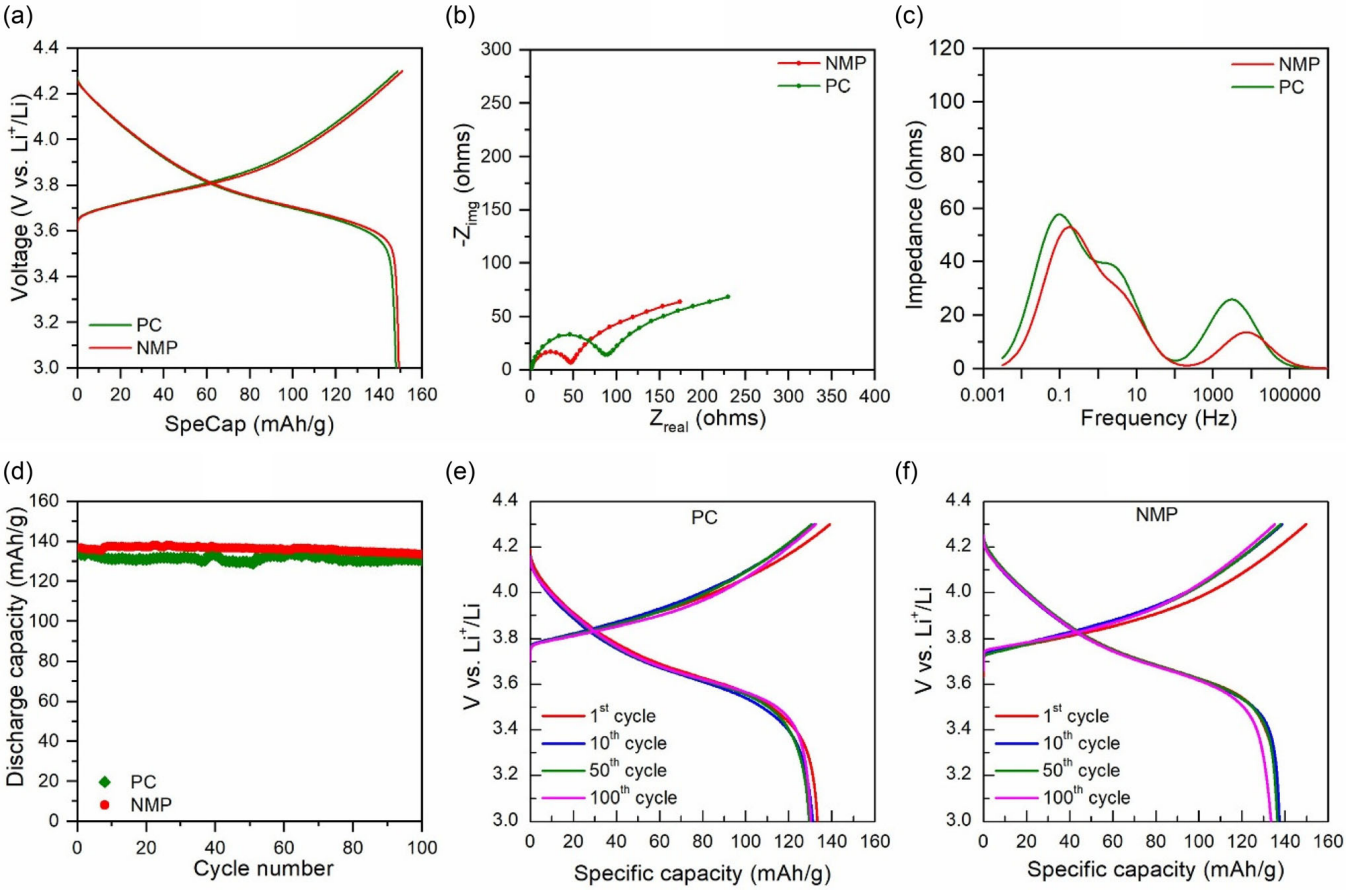
Electrochemical performance at high AM loading (94:3:3, AM/CB/PVDF, wt%) in half cells. a) Charge–discharge voltage profiles at the third formation cycle (C/10). b) Nyquist plots after formation. c) Corresponding DRT analyses highlighting impedance contributions. d) Long‐term cycling performance at C/3. e,f) Changes in charge–discharge voltage profiles during cycling at C/3.

Building on the promising results at low AM loading, we further evaluated the electrochemical performance of PC‐processed NMC electrodes at higher AM loading (94:3:3 composition) in half‐cell configurations. At a low C‐rate (C/10), the PC‐processed electrode delivered a discharge capacity of ≈148 mAh g^−1^, closely matching that of the NMP‐processed electrode (≈149 mAh g^−1^) (**Figure** **6**a), indicating that PC maintains comparable performance despite the increased AM content.

However, EIS analysis revealed an increase in the difference in overall resistance (39% for NMP vs. 80% for PC) at higher AM loading (Figure 6b). The PC‐processed electrode (≈87 ohms) exhibited a larger semicircle compared to the NMP‐processed electrode (≈47 ohms), reflecting a greater increase in the interfacial resistance relative to its NMP counterpart. DRT analysis (Figure 6c) provides deeper insight into this difference. The high‐frequency peak (>100 Hz) was more pronounced for the PC‐processed electrode, indicating increased CEI resistance and/or contact resistance at higher AM content. Higher AM loading in electrodes leads to a larger NMC‐electrolyte interfacial area, denser electrodes, reduced porosity, and higher tortuosity, thereby elevating CEI and contact resistance. This effect was more pronounced in the PC‐processed electrode.

Additionally, a newly distinct DRT peak in the frequency range of 1–10 Hz, absent in the low AM loading electrodes, appeared for both electrodes, with the PC‐processed electrode exhibiting a larger peak. As AM loading increases, electrode tortuosity rises, and local ionic depletion near particle surfaces becomes more pronounced, leading to slower charge transfer kinetics. This separation of time constants causes the charge transfer process to emerge as a distinct relaxation peak, independent of solid‐state Li^+^ diffusion.

The minor increase in charge transfer resistance observed for the PC‐processed electrode suggested greater resistance to ionic diffusion within the porous structure, likely associated with residual solvent effects. TGA analysis (Figure S9, Supporting Information) confirmed the presence of residual PC after drying, suggesting that solvent molecules may remain trapped within the electrode's porous structure. These residuals could impede ionic mobility and contribute to increased interfacial resistance. Although binder migration has previously been reported in systems with high‐viscosity solvents,^[^ ^45^, ^46^ ^]^ our cross‐sectional SEM imaging, along with EDX mapping (Figure S7, Supporting Information), revealed no evidence of binder stratification or surface accumulation under the tested conditions and electrode thickness.

However, it is worth noting that binder migration could still become a concern in thicker or denser electrodes, where solvent removal becomes more challenging and drying‐induced gradients are more likely to develop. Thus, future investigations should explore the interplay between drying profiles, electrode thickness, and binder distribution.

The low‐frequency peak (<1 Hz) remained comparable between the two electrodes, suggesting that intrinsic solid‐state Li^+^ diffusion within NMC particles was unaffected.

Long‐term cycling at C/3 (Figure 6d) demonstrated that both PC‐ and NMP‐processed electrodes maintained comparable capacity retention over 100 cycles. The PC‐based electrode retained 97.7% of its initial capacity, while the NMP‐based electrode maintained 97.6%. Both electrodes exhibited stable cycling performance with minimal capacity degradation. In contrast, previous studies on GVL‐based electrodes reported significant capacity fade (≈88%) under similar conditions, further highlighting PC as a more promising alternative.^[^ ^18^ ^]^

However, the PC‐processed electrode consistently showed a slightly lower reversible capacity at C/3. This discrepancy is attributed to higher overpotential (Figure 6e, f), reflecting increased polarization resulting from elevated interfacial resistance and restricted ionic diffusion within the porous structure. These challenges highlight the need for drying process optimization to ensure thorough and uniform removal of PC, thereby enhancing the performance of PC‐based electrodes at higher AM loadings and C‐rates, particularly in improving reversible capacity under more demanding conditions.

### 3.4 Electrochemical Performance of PC‐Processed NMC Electrodes in Full‐Cell Configurations

**Figure 7:**
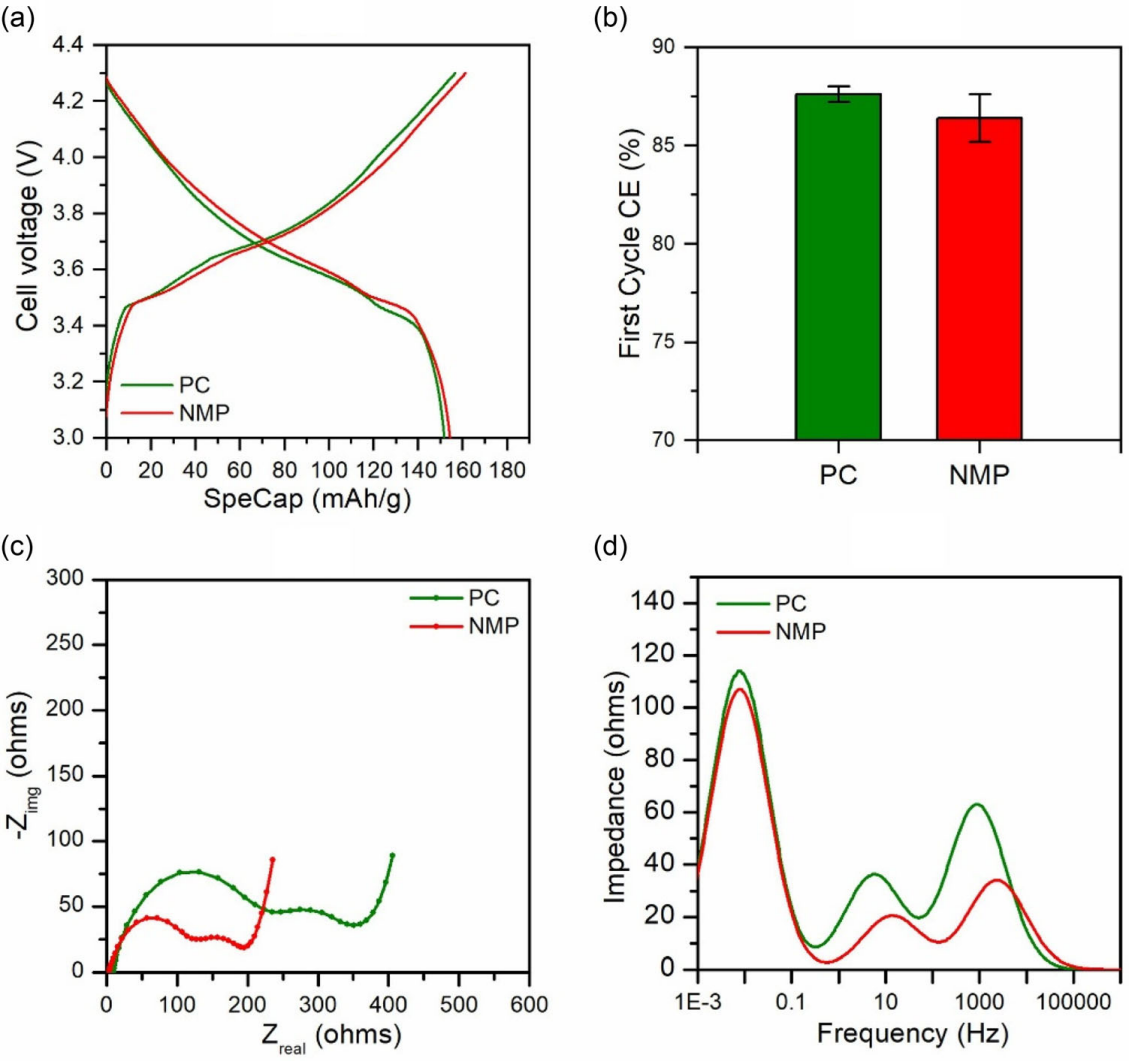
a) Charge–discharge voltage profiles at C/20 (third formation cycle) for PC‐ and NMP‐processed NMC/graphite full cells. b) First‐cycle CE for each cell. c) Nyquist plots after formation cycles showing initial impedance behavior. d) DRT spectra corresponding to impedance in (c), highlighting differences in interfacial and charge‐transfer resistances.

To assess the practical applicability of PC‐processed electrodes, we further evaluated NMC electrodes (94:3:3 composition) in full‐cell configurations with graphite anodes. The charge–discharge profiles at C/20 revealed nearly identical voltage curves and discharge capacities between PC‐ and NMP‐processed electrodes, indicating comparable initial electrochemical utilization (**Figure** **7**a). In line with the half‐cell observations, the first cycle CE of the PC‐processed electrode was slightly higher than that of the NMP counterpart (Figure 7b), suggesting reduced parasitic side reactions during early formation.

Despite comparable electrochemical performance during formation, EIS (Figure 7c) indicated higher overall resistance in the PC‐based full cell. DRT analysis (Figure 7d) revealed that both electrodes exhibited similar low‐frequency (<0.1 Hz) impedance features, which are typically associated with solid‐state Li^+^ diffusion limitations in a fully lithiated NMC cathode. As EIS measurement was performed at 0% state of charge and both cells used the same graphite anode and electrolyte, the low‐frequency peak is primarily attributed to Li^+^ diffusion within the NMC cathode, where vacant intercalation sites are scarce. The more pronounced difference appeared in the mid‐to‐high frequency regions. The high‐frequency peak, representing surface film and contact resistance, and the mid‐frequency peak, associated with charge‐transfer processes at the electrode–electrolyte interface, were both elevated in the PC‐processed electrode. Given the identical graphite and electrolyte conditions, this suggests that the interfacial characteristics of the cathode, likely influenced by processing, account for the observed impedance differences. Nevertheless, these differences had a minimal impact on low‐rate performance, as evidenced by overlapping voltage profiles and similar polarization during formation. Overall, these results indicate that PC‐based processing does not compromise the initial reversible capacity or CE of the electrode.

**Figure 8:**
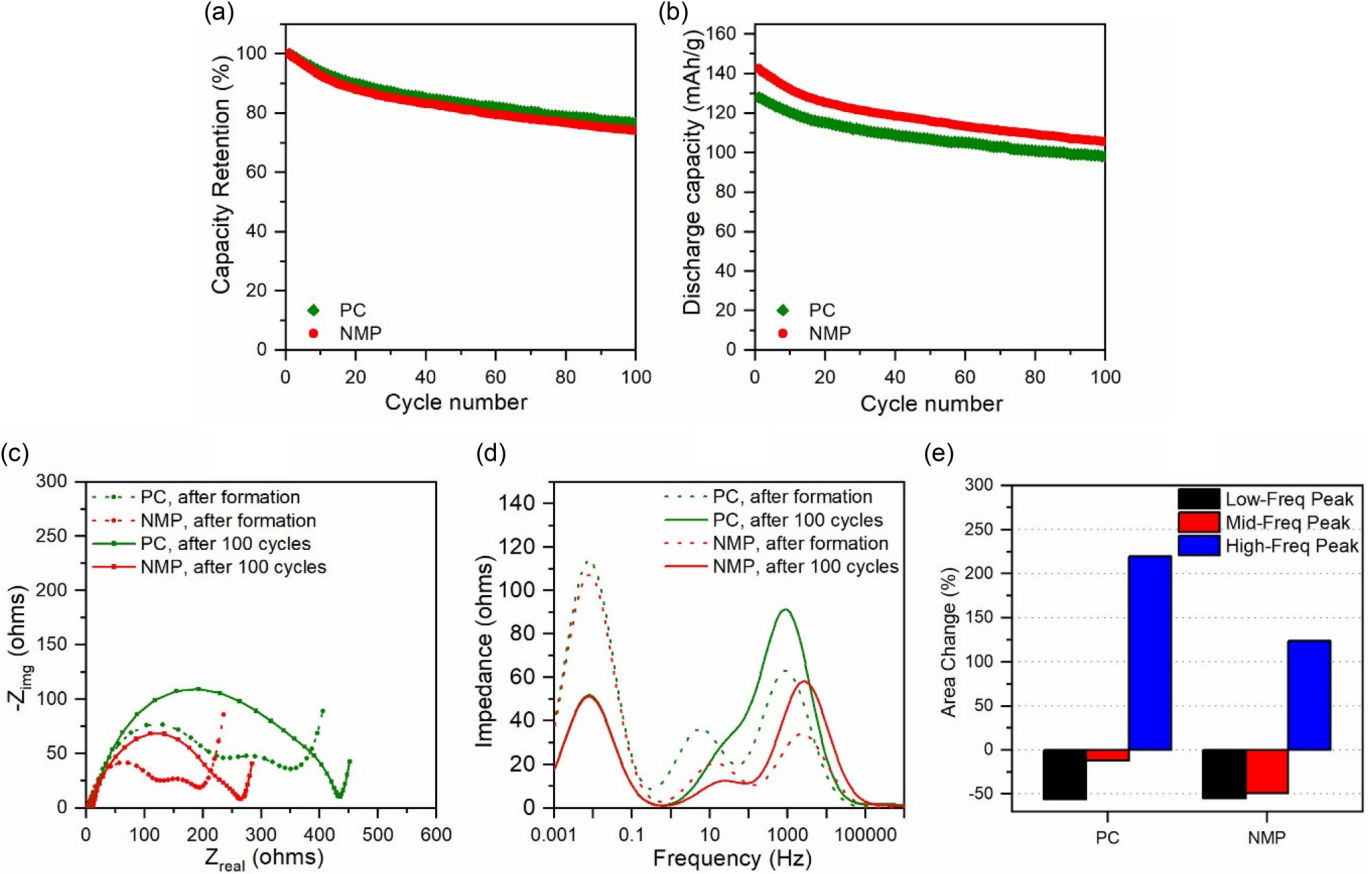
a) Capacity retention over 100 cycles at C/3 for PC‐ and NMP‐processed NMC/graphite full cells. b) Discharge capacity over 100 cycles at C/3. c) Comparison of Nyquist plots after 100 cycles. d) Comparison of DRT spectra showing impedance contributions. e) Quantified change in DRT peak areas, illustrating greater increases in high‐frequency impedance for the PC‐processed electrode.

Long‐term cycling performance at C/3, however, revealed rate‐dependent limitations. As shown in **Figure** **8**a, and 8b, both full cells retained ≈80% of their initial capacity after 100 cycles, but the PC‐processed electrode consistently delivered lower discharge capacity than the NMP‐processed one. This disparity was not evident at low C‐rates but emerged with prolonged moderate‐rate cycling. EIS data collected after 100 cycles (Figure 8c) showed a larger increase in overall resistance for the PC‐based cell, particularly in the high‐frequency region. DRT spectra (Figure 8d) further confirmed a more pronounced growth of the high‐frequency peak in the PC‐based cell, indicative of increased surface film resistance and possible degradation of interparticle or aluminum‐particle contacts. Quantitative analysis (Figure 8e) indicated that the PC‐based cell exhibited over twice the relative increase in the high‐frequency peak compared to the NMP‐based cell.

These results collectively suggest that the rate‐dependent performance difference between PC‐ and NMP‐processed electrodes stems from interfacial and contact resistance, rather than from initial inventory loss or intrinsic material limitations. The impedance growth—primarily in the high‐frequency domain—likely originates from the growth of the resistive CEI layer and possible loss in electronic contact within the cathode matrix. These effects remain largely dormant under slow cycling conditions but become limiting at higher rates.

Importantly, the observed performance gap is pronounced under high AM loading and was not evident in low‐loading electrodes. We attribute the rate‐dependent behavior to the presence of residual PC solvent, which may not have been completely removed during cycling. Incomplete solvent evaporation can interfere with cathode interfacial stability, promoting SEI growth and increasing resistance at the particle–binder or particle–current collector interfaces. However, this limitation is not fundamental to the PC‐based process and can be mitigated through careful optimization of electrode drying. In particular, the implementation of a multizone heating profile with staged temperature control could enhance solvent removal and improve binder distribution, thereby minimizing interfacial resistance buildup.^[^ ^19^, ^37^, ^47^ ^]^ With such process refinements, PC‐based electrode fabrication remains a promising route for achieving sustainable and high‐performance LIB cathodes, even under high‐loading conditions.

## 4 Conclusions

This study provides a comprehensive and systematic evaluation of PC as a sustainable solvent for LIB cathode manufacturing. While the solubility of PVDF in PC has been previously acknowledged, this work is, to the best of our knowledge, the first to validate PC's practical processability across a full electrode fabrication workflow under process‐relevant conditions, including evaluation in both half‐cell and full‐cell configurations, using multiple cathode chemistries and a range of AM loadings. The results demonstrate PC's compatibility with PVDF binders and its potential to support ecofriendly electrode fabrication with minimal compromise in performance.

Compared to NMP and other green solvent candidates, PC offers a favorable combination of low toxicity, non‐HAP classification, biodegradability, lower cost, and nonvolatile behavior, making it attractive for industrial‐scale applications. In addition to its environmental and safety benefits, PC is commercially available at a lower cost per liter than NMP. Its benign classification significantly reduces operational costs associated with handling, ventilation, and worker safety infrastructure. Although PC's higher boiling point increases the energy demand for drying, this could be effectively mitigated through further drying process engineering.

This work further demonstrates that PC‐based slurries exhibit consistent rheological behavior and uniform coating quality, leading to successful cathode fabrication with strong adhesion to the current collector. Importantly, all processing steps were performed using conventional mixing and coating equipment with minor process modifications, underscoring the scalability and practical integration of PC‐based systems into existing manufacturing lines.

Electrochemical evaluations revealed that PC‐based electrodes exhibit excellent initial CE and comparable performance to NMP‐based electrodes at lower AM loadings. At higher loadings and elevated C‐rates, PC‐based electrodes showed increased interfacial and contact resistance, attributed primarily to residual solvent effects, as confirmed by TGA analysis. These results highlight the importance of drying process optimization to enhance interfacial resistance and minimize performance loss, especially in thicker or denser electrodes.

Future efforts should focus on refining drying protocols—such as implementing multizone heating profiles or real‐time solvent monitoring—to ensure complete solvent removal and uniform binder distribution. By bridging the gap between fundamental solvent characteristics and practical manufacturing constraints, this study establishes a strong foundation for the industrial adoption of PC as a green and effective alternative to NMP, offering a path toward safer and more environmentally responsible LIB manufacturing.

## Conflict of Interest

The authors declare no conflict of interest.

## Supporting information

Supplementary Material

## Data Availability

The data that support the findings of this study are available from the corresponding author upon reasonable request.
