# Supplementary material for: Exploring Propylene Carbonate as a Green Solvent for Sustainable Lithium‐Ion Battery Cathode Manufacturing
Source: ChemSusChem. 2025 Aug 20;18(19):e202500937. doi: 10.1002/cssc.202500937 (PMC12487736; doi:10.1002/cssc.202500937)
Supplement: Supplementary file 1 — Supplementary Material [file CSSC-18-e202500937-s001.pdf]

Supporting Information (SI)

Exploring Propylene Carbonate as a Green Solvent for Sustainable Lithium-ion Battery Cathode Manufacturing

Author Names: Mazedur Rahman, Hosop Shin[Z]

Affiliation(s): Purdue University, Indianapolis, IN 46202, United States

[z] Corresponding Author E-mail Address: shin282@purdue.edu

Number of pages: 13

Number of figures: 10

Number of videos: 1

Number of tables: 2

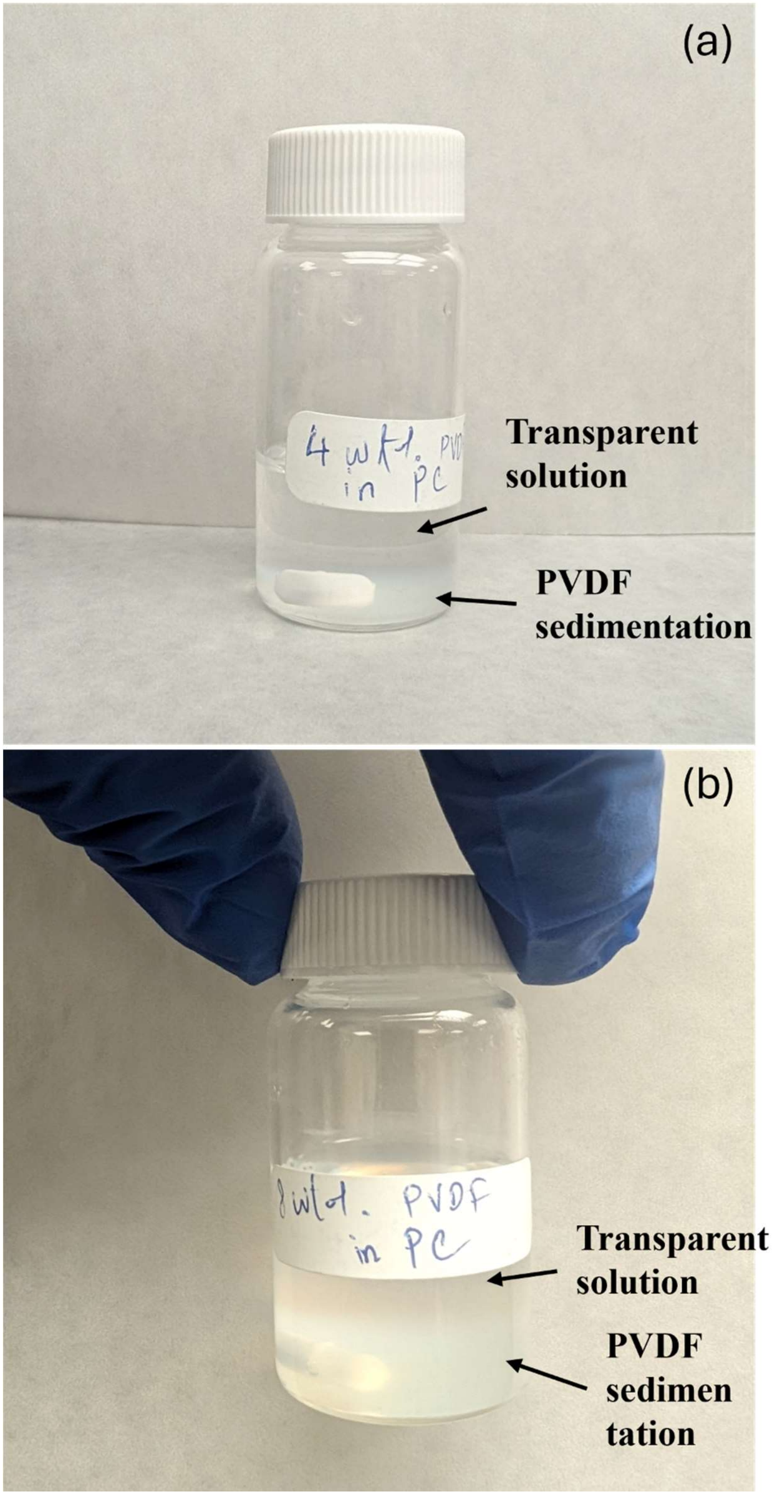

Figure S1. PVDF binder mixed in PC at 80°C for 24 hours using magnetic stirring on a hotplate: (a) 4wt% and (b) 8 wt%.

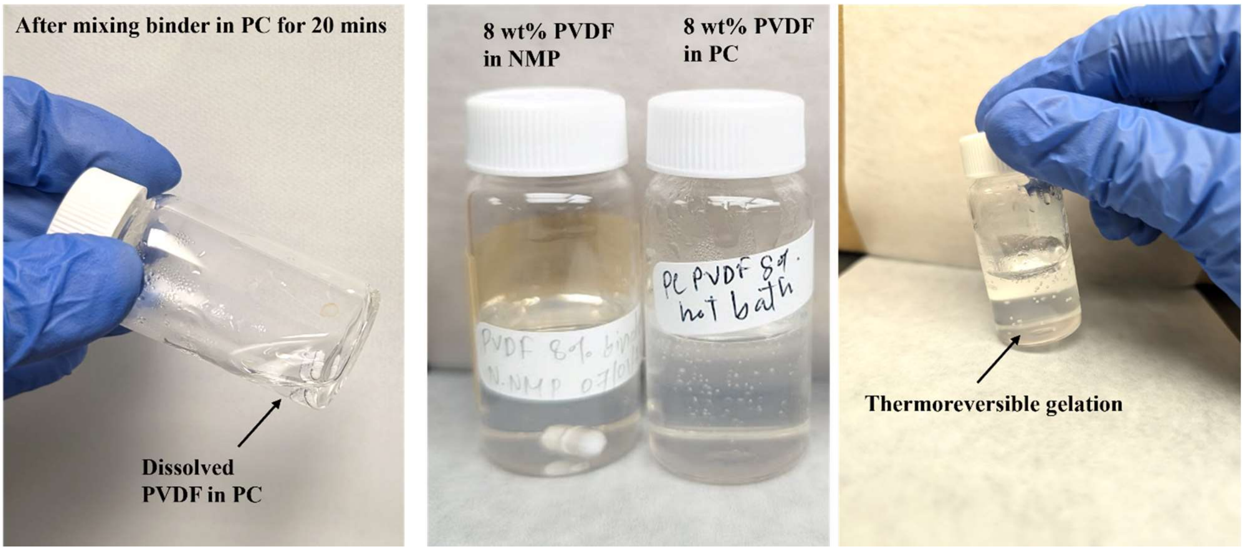

Figure S2. PVDF dissolution in PC by heating with a hot water bath enclosure.

Table S1. Measured viscosity values as a function of rotational speed for PC-based and NMP-based cathode slurries

Rotational speed (RPM)	Shear rate (1/s)	Viscosity of PC-based slurry	Viscosity of NMP-based slurry
6	0.80	1.96	1.23
12	1.61	1.18	0.82
30	4.02	0.77	0.50
60	8.04	0.43	0.36

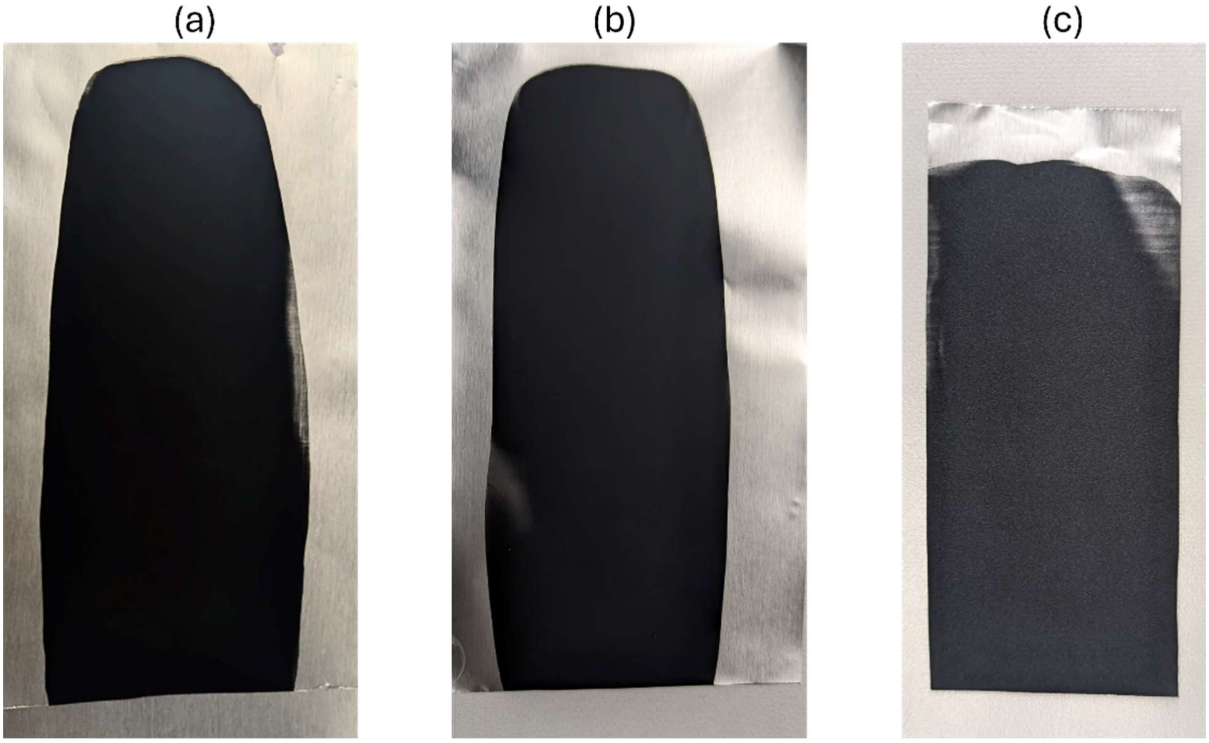

Figure S3. Surface morphology of dried electrodes prepared with 94% active materials (a, b: NMC, c: LCO) using (a,c) PC-based and (b) NMP-based binder solutions after slurry coating.

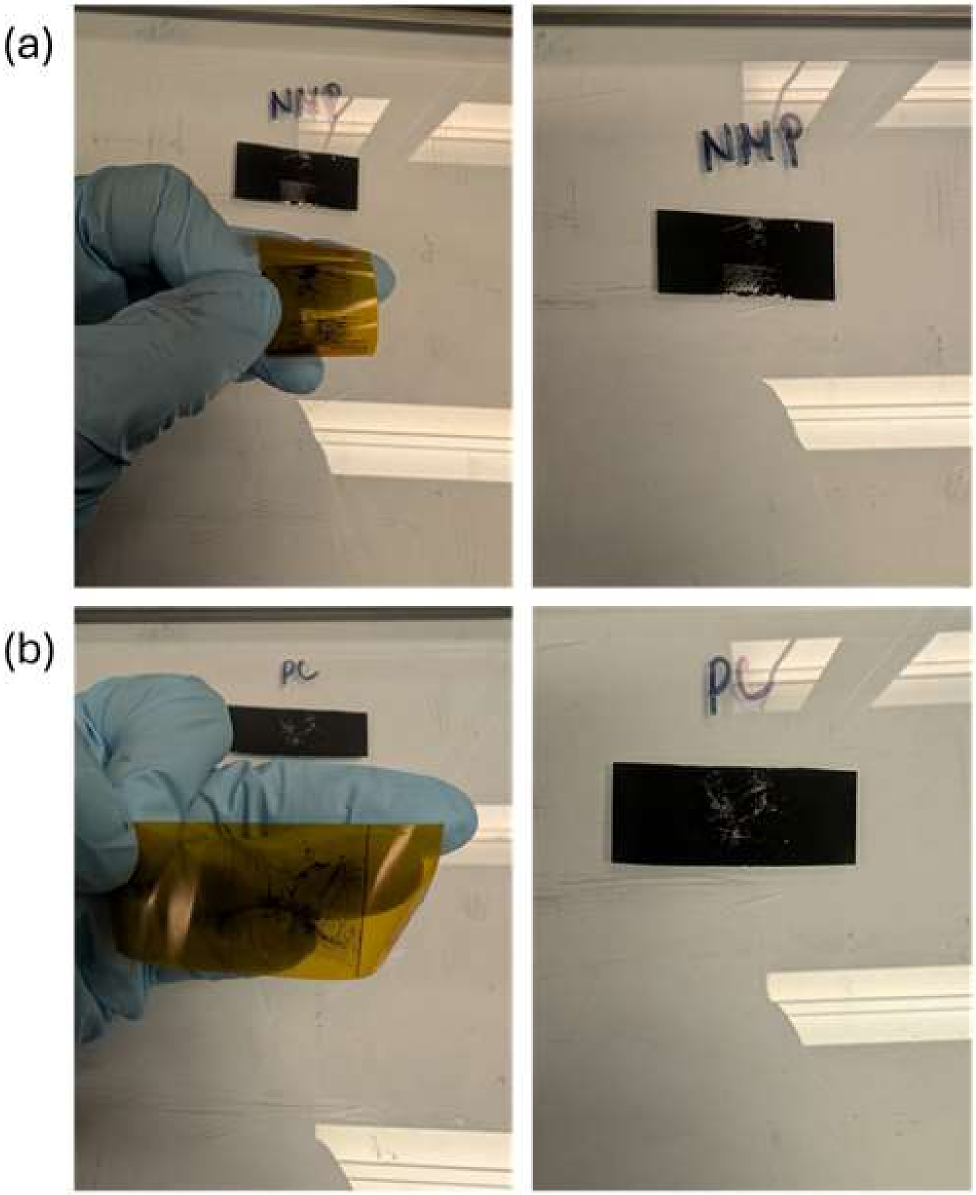

(c)

	Electrode weight before peeling test	Electrode weight after peeling test	Peeling percentage
PC	0.144 g	0.139 g	3.47%
NMP	0.121 g	0.115 g	4.96%

Figure S4. Photographs of the Kapton tape peel test for (a) NMP-processed and (b) PC-processed electrodes, along with (c) quantification of material detachment after the test for each electrode.

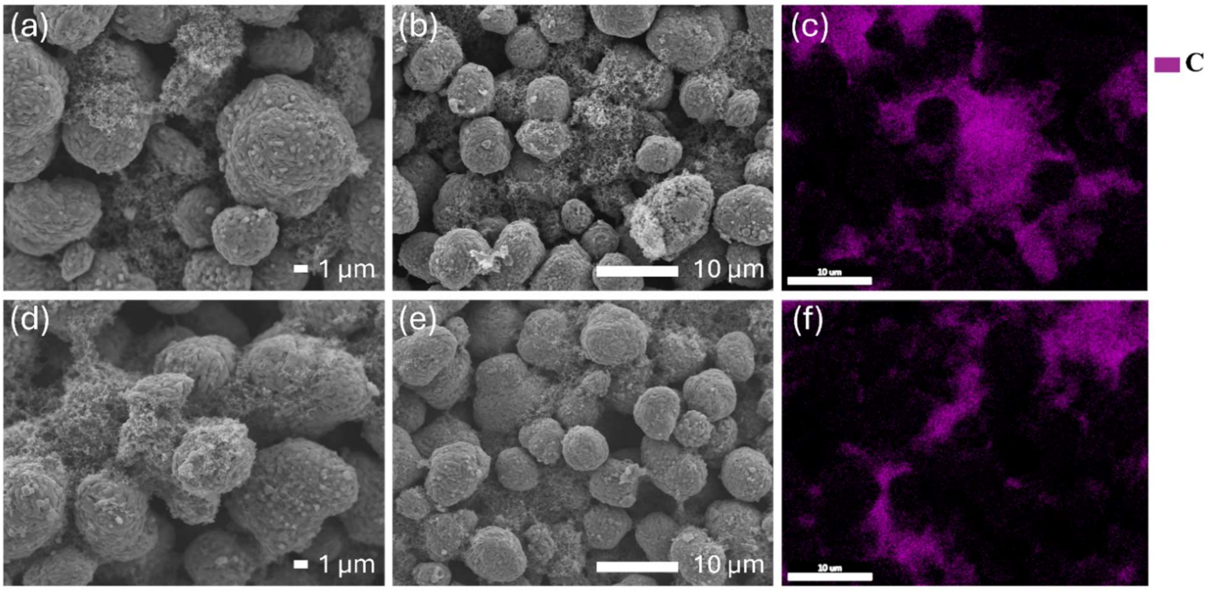

Figure S5. SEM images and corresponding EDAX mapping of carbon network of dried NMC electrodes prepared using (a-c) PC-based and (d-f) NMP-based binder solutions.

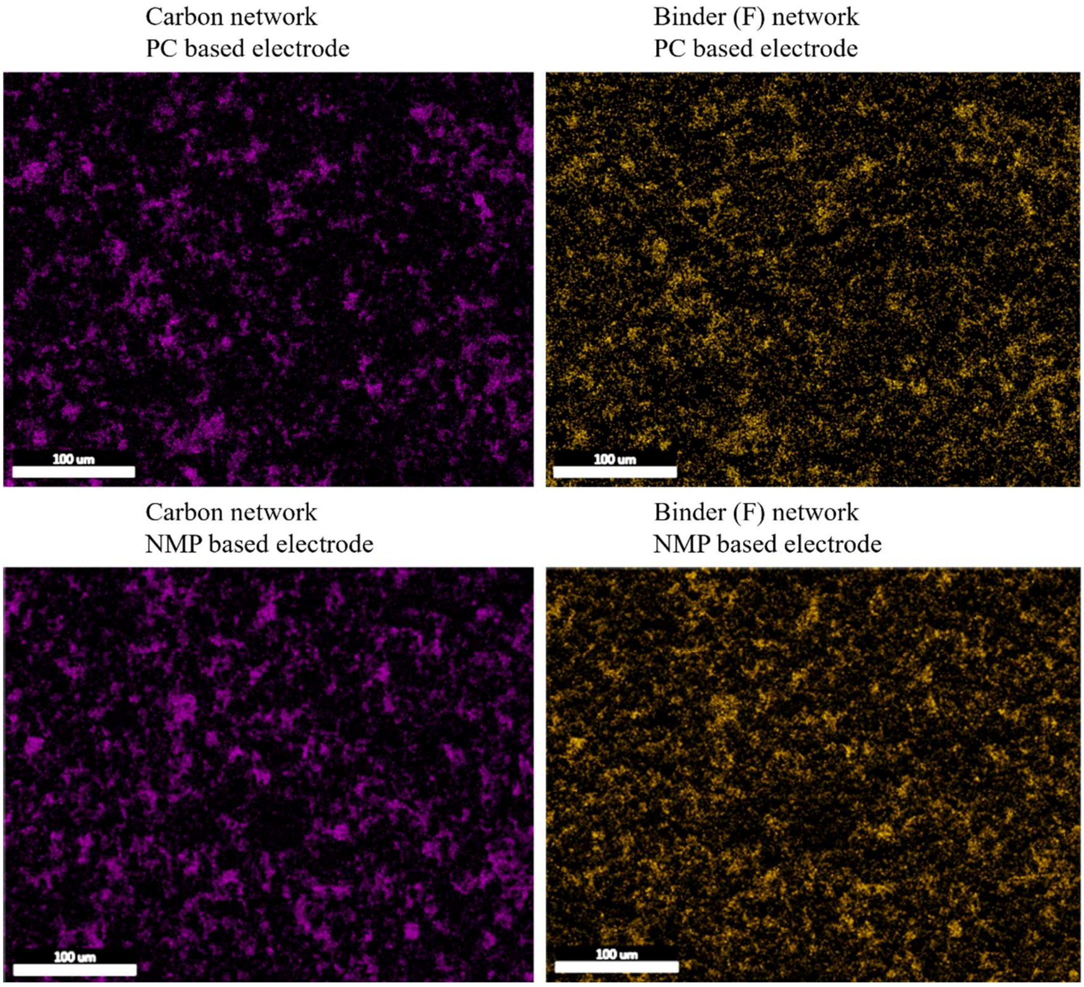

Figure S6. EDAX elemental mapping illustrating the distribution of conductive carbon and binder (F) networks on the surface of dried NMC electrodes prepared by PC- and NMP-based binder solutions.

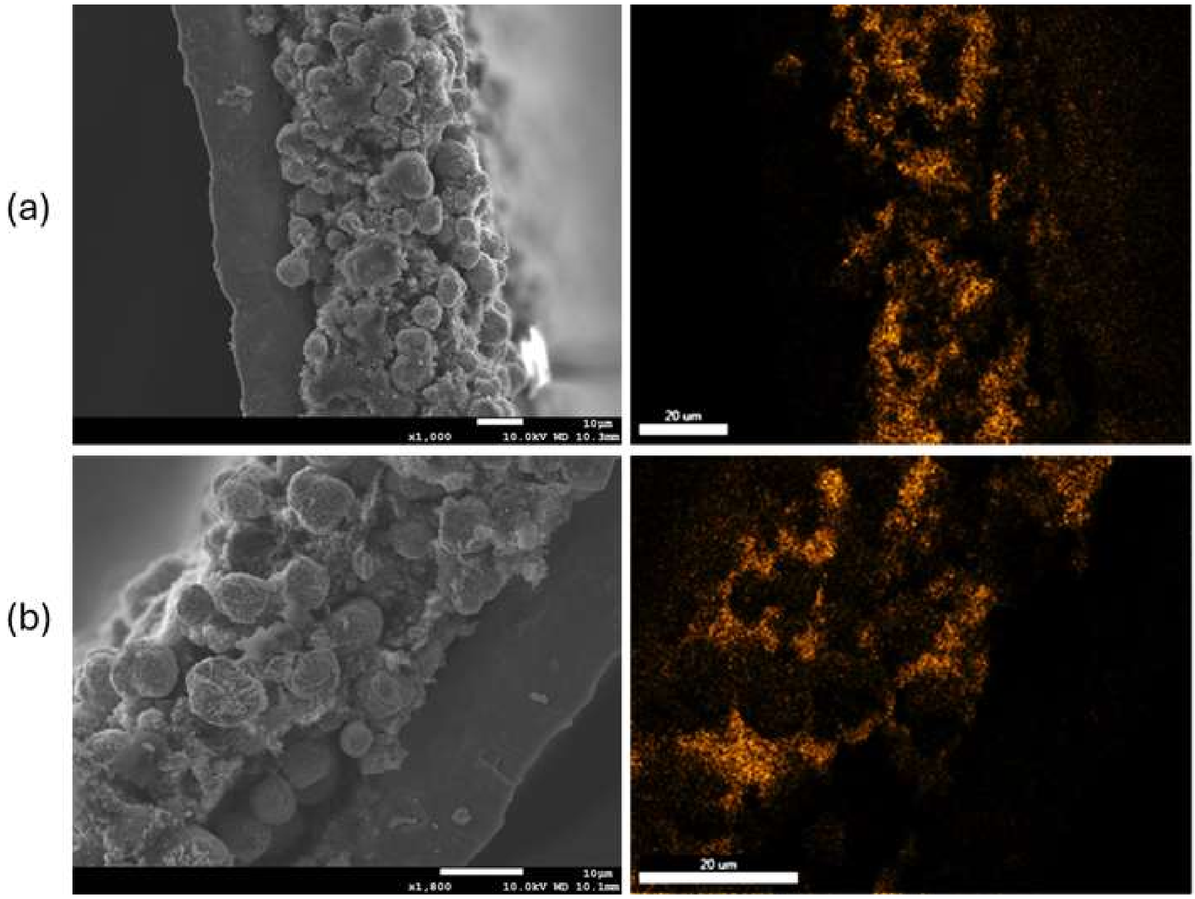

Figure S7. Cross-sectional SEM and corresponding EDX mapping of fluorine (F) for (a) PC-processed and (b) NMP-processed electrodes with 85% active material.

Table S2. Electrode information of NMC111 electrodes (85% AM)

Solvent	Cathode	Composition	1st Cycle C.E.	Standard Deviation	Reference
PC	NMC111	85:7.5:7.5	87.99	0.5	This work
NMP	NMC111	85:7.5:7.5	79.37	3.24	This work
PC	NMC111	94:3:3	87.83	1.41	This work
NMP	NMC111	94:3:3	85	2.78	This work
TEP	NMC622	94:3:3	86.6	-	[12]
CYRENE	NMC811	90:5:5	82.2	-	[17]
Polarclean	NMC111	80:10:10	70.15	-	[19]

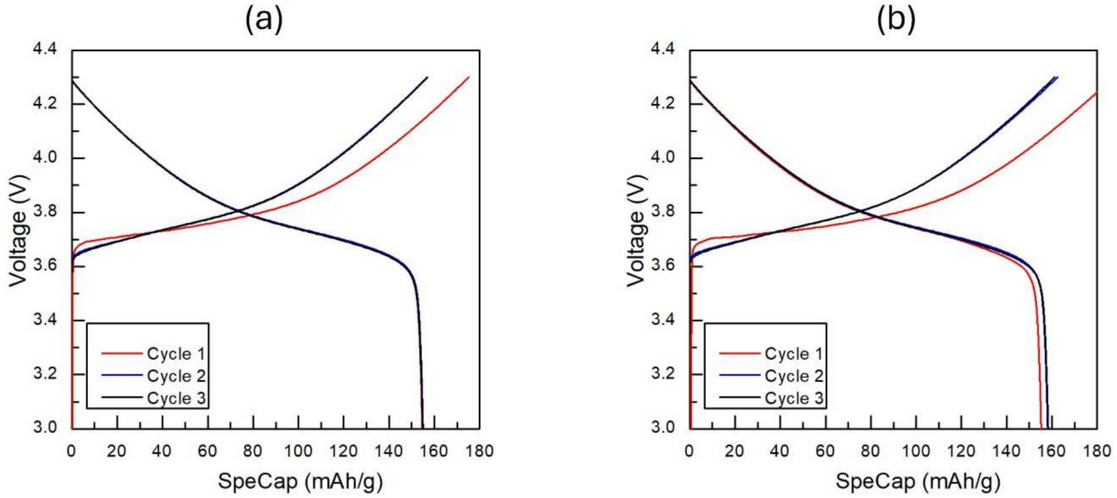

Figure S8. Charge-discharge voltage profiles of (a) PC- and (b) NMP-processed NMC electrodes during the formation cycles at C/10.

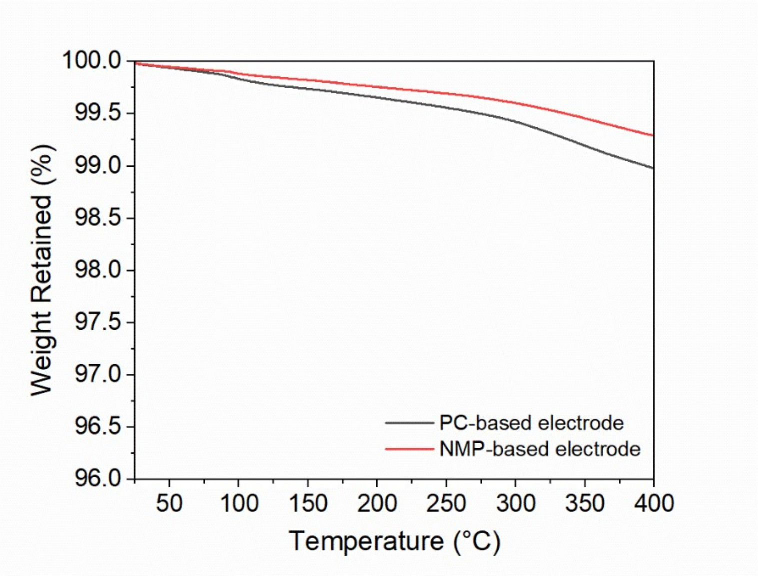

Figure S9. TGA profiles of PC- and NMP-processed NMC electrodes. A small amount of electrode material was collected by gently peeling off the surface layer for TGA analysis.

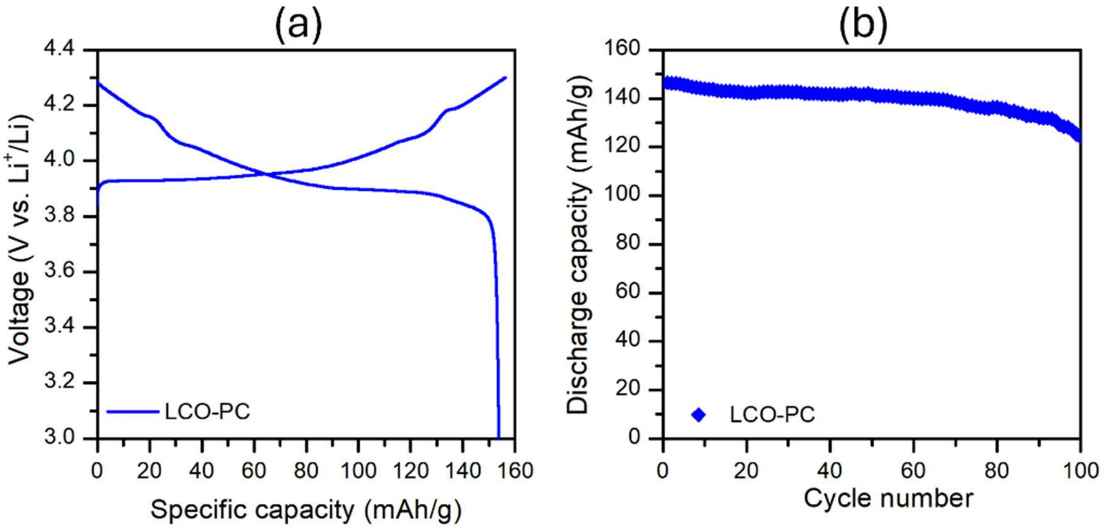

Figure S10. a) Charge-discharge curves of LCO half cells (94% AM) after the formation cycles (3 cycles) at C/10 rate and b) long-term cycling at C/3 rate.
